# Beta-tricalcium phosphate combined with native bone proteins (β-TCP - NBP): a novel bone graft substitute for ankle and hindfoot arthrodesis

**DOI:** 10.1007/s00264-025-06429-z

**Published:** 2025-02-04

**Authors:** Juhana Leppilahti, Mari Kuoppala, Timo Sirola, Lukasz Kolodziej, Katri Ahonen, Mikko Aulamo, Jaakko Niinimäki, Pekka Jalovaara

**Affiliations:** 1 Translational Medicine Research Unit, Medical Research Center Oulu, Oulu University Hospital and University of Oulu, Oulu, Finland; 2 Department of Orthopaedics and Traumatology, University of Helsinki and Helsinki University Hospital, Helsinki, Finland; 3 Orthopaedic Traumatology and Orthopaedic Oncology Clinic SPSK No 1, Pomeranian Medical University, Szczecin, Poland; 4 Central Hospital of Central Finland, Jyväskylä, Finland; 5 South Karelia Central Hospital, Lappeenranta, Finland; 6 Department of Radiology, Faculty of Medicine, University of Oulu, Oulu, Finland

**Keywords:** Bone graft substitute, Ankle fusion, Hindfoot fusion, Nonunion, Bone growth factors, Osteoinduction, Beta-tricalcium phosphate, Pain relief, Function of ankle and hindfoot

## Abstract

**Purpose:**

The purpose of this prospective, multi-centre study was to assess the performance and safety of a combination of osteoconductive β-tricalcium phosphate and osteoinductive native bone proteins (β-TCP - NBP) used as alternative for autograft in ankle and hindfoot arthrodesis.

**Methods:**

Thirty-four patients enrolled underwent ankle or hindfoot arthrodesis with β-TCP - NBP and were evaluated radiographically, clinically, and functionally up to fifty-two weeks. The primary performance endpoint was fusion rate evaluated with CT at six months. Safety was assessed based on the severity and incidence of adverse events. Functional evaluation was performed using the American Orthopaedic Foot & Ankle Society (AOFAS) ankle-hindfoot score and pain was recorded using the Visual Analogue Scale (VAS).

**Results:**

CT at 6 months showed that 85.3% had osseous bridging of the joint of ≥ 25%, 52.9% ≥50%, 8.8% <25% and 5.9% showed no bridging. The AOFAS score increased significantly from 60.4 ± 17.6 points at operation to 68.6 ± 17.2 points at six months and to 73.5 ± 17.7 points at 12 months. The group with fusion rate ≥25% showed significantly higher AOFAS score than that with fusion rate < 25% at 12 months. The mean VAS pain score at rest and during weight bearing decreased significantly (*p* < 0.0001) from operation to six and 12 months.

**Conclusion:**

This study demonstrated that β-TCP - NBP is a valuable bone graft substitute for fusion of ankle and hindfoot due to debilitating osteoarthritis and offers an alternative for autograft.

**Level of evidence:**

Level II.

## Introduction

Patients with end-stage foot and ankle arthritis have limited physical function, lower health-related quality of life and severe pain [1]. A common surgical treatment for these patients is arthrodesis [2, 3]. Nonunion after arthrodesis remains a concern [4], and therefore autograft is commonly used to enhance bony fusion [5]. However, harvesting of autograft is associated with increased operation theatre time and donor site morbidity and complications, such as pain, infection, and fracture [6, 7]. Therefore, bone surgeons await an ideal, ready-to-use substitute for autograft [8]. Typically, used bone graft substitutes are based solely on osteoconductive ceramic materials [9]. In clinical practice, only a few bone graft substitutes leverage the ancillary effects of growth factors, such as osteoinductivity or osteostimulativity, to enhance the function of osteoconductive ceramic components [10–12].

β-TCP - NBP is an osteoconductive and osteoinductive bone graft substitute. The idea behind β-TCP - NBP is to mimic natural bone, as it contains calcium phosphate granules resembling bone mineral and bone proteins extracted from the long bones of reindeer. The intended action of bone healing is achieved by the osteoconductive β-TCP matrix assisted by the ancillary osteoinductive action of bone proteins including bone growth factors, and thus approaching the mechanism and performance of natural bone [13, 14].

The goal of this prospective, multi-centre clinical study was to assess the performance and safety of β-TCP– NBP used as a bone graft substitute in ankle and hindfoot arthrodesis for primary or secondary post-traumatic osteoarthritis.

## Materials and methods

A prospective clinical trial was undertaken during 2013–2017 at five clinical sites. Included were skeletally mature (> 18 years of age) independent, cooperative, ambulatory patients requiring ankle or hindfoot fusion due to primary or secondary post-traumatic osteoarthritis. Exclusion criteria were the following: radiographic evidence of bone defects, untreated malignant neoplasm at the surgical site, history of alcohol/drug abuse within 12 months and/or tobacco use within six months, medication or diseases known to affect the skeleton or the immune system, patients with physically or mentally compromised health, those with prior ankle or hindfoot fusion surgery requiring more than three screws or other-than-screw fixation or those who were pregnant. The trial was prospectively registered at Clinicaltrials.gov (NCT02480868). This study was performed in line with the principles of the Declaration of Helsinki. Local ethics committees approved the study, and patients signed an independent ethics committee approved informed consent form prior to enrollment.

All fusions were supplemented with β-TCP - NBP bone graft substitute consisting of a combination of β-tricalcium phosphate granules and bone proteins of reindeer origin including bone growth factors in a ready-to-use 3 cc syringe in paste form.

### Surgical technique

The arthrodesis procedure of the ankle and hindfoot joints included routine removal of the cartilage and rigid internal fixation with two to three screws across the fusion line. In ankle fusion, a bed of 2.5 × 0.5 × 0.7 cm was created over the anterior fusion line medially or laterally. The bed was filled with β-TCP– NBP paste, and the rest of the paste was introduced between the fusion surfaces. In the fusion of the talocalcaneal joint, the β-TCP– NBP paste was introduced between the fusion surfaces and in the 2.5-cm-deep, 6 mm-diameter horizontal drill-hole created through the fusion line at the distal edge of the proximal facet. Half of the hole was on the calcaneal side and half on the talar side. Postoperatively, orthosis or a plaster cast was used for six weeks (changed at two weeks), and a walking cast or orthosis was used for a further six weeks to immobilize the ankle. Non-steroidal anti-inflammatory painkillers were contraindicated three months postoperatively.

### Outcome measures

**Fig. 1:**
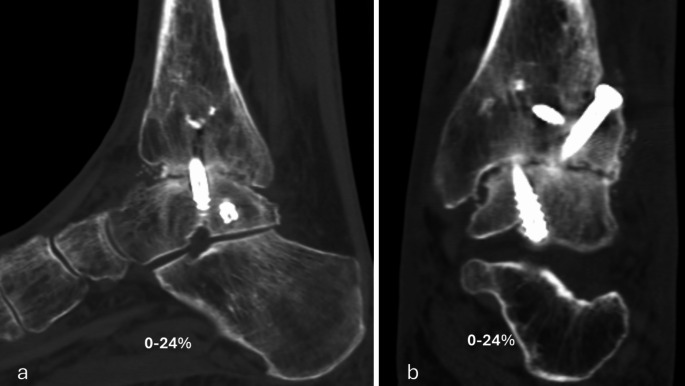
Six months lateral (**a**) and anteroposterior (**b**) CT scan of the ankle arthrodesis showing < 25% fusion rate

**Fig. 2:**
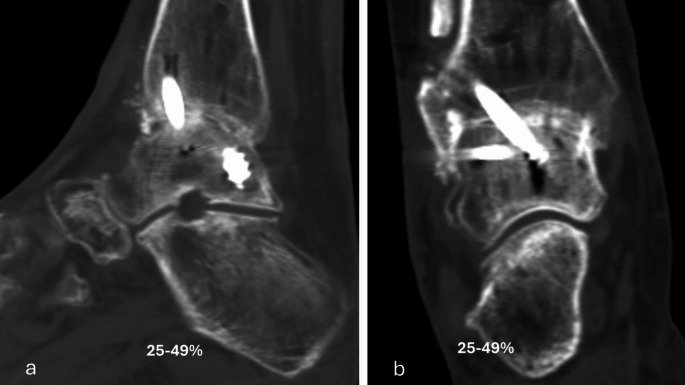
Six months lateral (**a**) and anteroposterior (**b**) CT scan of the ankle arthrodesis showing 25–50% fusion rate

**Fig. 3:**
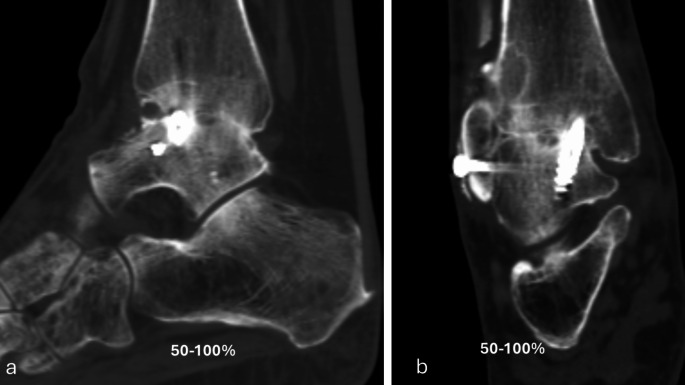
Six months lateral (**a**) and anteroposterior (**b**) CT scan of the ankle arthrodesis showing > 50% fusion rate

**Fig. 4:**
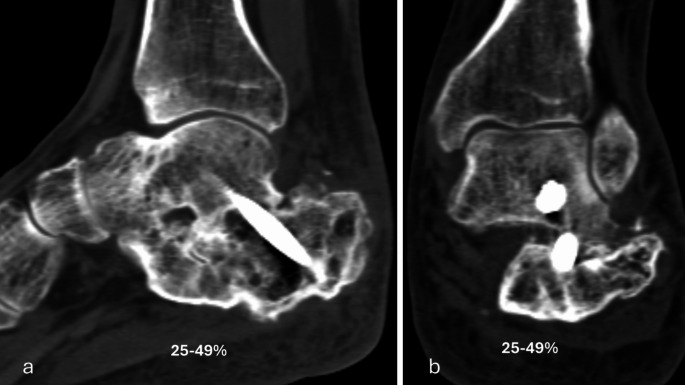
Six months lateral (**a**) and anteroposterior (**b**) CT scan of the subtalar arthrodesis showing 25–50% fusion rate

**Fig. 5:**
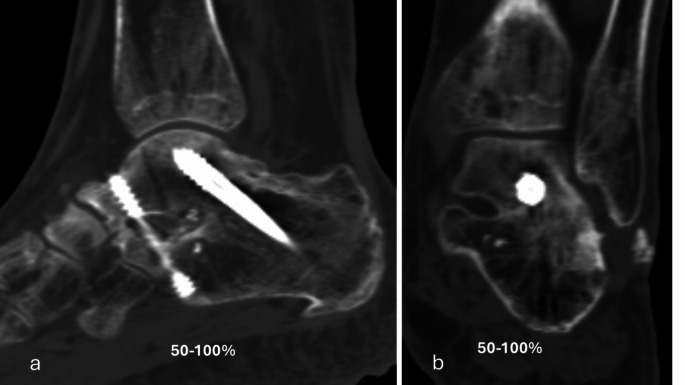
Six months lateral (**a**) and anteroposterior (**b**) CT scan of the subtalar arthrodesis showing > 50% fusion rate

The primary performance endpoint was fusion rate evaluated with CT at six months. Percent of osseous bridging was evaluated on the basis of benchmarks of 0–24%, 25–49% and 50–100% (Figs. 1, 2, 3, 4 and 5) [11]. Additionally, joints that had osseous bridging of 0–24% were re-reviewed and categorized as bridging over the fusion line or no bridging. Clinical evaluations were performed preoperatively and at two, six and 12 weeks and at six and 12 months. At all visits, standardized radiographs were taken and a joint was considered fused if the medial, lateral, posterior, or anterior joint line was evaluated as fused. The radiographic images were assessed independently by two trained radiologists. In cases of disagreement, consensus was reached in review meetings. Inter-rater reliability assessment was conducted for the osseous bridging. Weighted kappa coefficient was 0.77 (95% CI 0.67; 0.87) which is considered as substantial agreement.

Functional evaluation was performed by using the non-validated American Orthopaedic Foot & Ankle Society (AOFAS) ankle-hindfoot score [15] at operation and at six and 12 months postoperatively, with scores ranging from 0 to 100 points and higher scores indicating greater functional performance. The time point for returning to work was recorded at six and 12 months. Pain was recorded using the Visual Analogue Scale (VAS) [16] at operation and at six and 12 months on a scale from 0 to 100 mm, with 0 representing “no pain” and 100 mm representing “worst imaginable pain” and at clinical examination at every follow-up as no pain, mild pain, moderate pain or severe pain. Radiographic union, clinical and functional evaluation served as secondary performance endpoints.

Safety was assessed in accordance with the ISO14155 guideline based on severity and incidence of adverse events and their relationship to β-TCP - NBP. Treatment emergent adverse events are adverse events occurring during or following treatment throughout the study. An adverse event was classified as serious if it led to death, a life-threatening illness or injury, a permanent impairment of a body structure or a body function, in-patient or prolonged hospitalisation or medical or surgical intervention to prevent the above-mentioned (ISO14155). Therapeutic failures were patients with nonunion or requiring secondary therapeutic intervention for delayed or nonunion [11].

### Statistical analysis

Values are presented as mean±standard deviation (SD). Change from operation to six and 12 months in AOFAS, VAS in rest and weight bearing and clinical assessment of pain were analysed with repeated measures analysis of covariance (RM-ANCOVA) models, with visit as a fixed effect, results at operation as a baseline covariate and patient as a random effect. An unstructured covariance structure was assumed. *P*-values < 0.05 were considered statistically significant. To further evaluate the characteristics of the < 25% osseous bridging group, several demographic and baseline characteristics and their impact on osseous bridging were investigated. For each variable a separate logistic regression model was fitted to model the odds to be in < 25% osseous bridging group using the variable in question as a fixed effect in the model. The results are compared in the Discussion with relevant studies using autograft, especially the article of DiGiovanni et al. [11] which used a similar study setting and design.

## Results

Thirty-four patients (mean 59.9 yrs, range 25.3–77.2 yrs, 18 females, 16 males) having primary (*n* = 9) or secondary, post-traumatic (*n* = 25) osteoarthritis were included in this study. Seven patients had cardiac, eight had vascular (hypertension) and ten had metabolic disorders (e.g. hypercholesterolaemia and diabetes mellitus). One patient had a blood system disorder, six had endocrine (hypothyroidism) and three had nervous system disorders and one had breast cancer and was undergoing hormonal therapy. Twenty patients (58.8%) were non-smokers, 14 (41.2%) were ex-smokers, 12 (35.3%) were non-drinkers and 22 (64.7%) had an average alcohol consumption. Thirteen (38.2%) patients had an arthroscopic and 21 (61.8%) an open surgery.

Based on CT evaluation at six months, 32 (94.1%) joints showed osseus bridging > 0% (Table 1). 85.3% of the joints showed osseus bridging ≥25% (Figs. 2 and 4). and 52.9% of the joints ≥50% (Figs. 3 and 5). Three cases exhibited a fusion rate of less than 25% (Fig. 1), showing only faint bridging. Two cases (5.9%) were classified as non-unions. None of these cases required reoperation for fusion-related issues during the study. No patient characteristics that would predict the < 25% osseous bridging were identified. Radiographic evaluation showed fusion in 90.6% of the joints at six months (two of the patients had missing data) and in 81.8% of the joints at 12 months (one of the patients had missing data, Table 1).

**Table 1:** Summary of radiographic evaluation of fusion

	Scale of the CT evaluation	Fusion rate % (*n*)
Primary endpoint: CT at 6 months (*n* = 34)	> 0%	94.1% (32)
	≥25	85.3% (29)
	≥50%	52.9% (18)
	Failure rate (nonunion)	5.9% (2)
Secondary endpoint: X-ray at 6 months (*n* = 32)		90.6% (29)
Secondary endpoint: X-ray at 12 months (*n* = 33)		87.9% (29)

The AOFAS score increased significantly from 60.4 ± 17.6 at operation to 68.6 ± 17.2 points at six months (*p* = 0.0186) and to 73.5 ± 17.7 points at 12 months (*p* = 0.001). The group with fusion rate ≥25% showed significantly higher AOFAS score than that with fusion rate < 25% at 12 months (*p* = 0.0376).

The mean VAS pain score at rest decreased significantly from 26.1 ± 21.8 at operation to 8.4 ± 11.4 at 6 months (*p* < 0.0001) and to 8.1 ± 11.8 at 12 months (*p* < 0.0001), and at weight bearing from 55.6 ± 26.4 to 32.4 ± 28.5 (*p* < 0.0001) and 22.2 ± 24.9 (*p* < 0.0001), respectively. The group showing fusion rate < 25% had significantly higher VAS pain score at weight bearing than the group of fusion rate over 25% at six months (*p* = 0.0018) and at 12 months (*p* = 0.0415).

In the clinical evaluation, at operation three patients (8.8%) had no pain, eight (23.5%) had mild pain, 17 (50.0%) had moderate pain, six (17.6) patients had severe pain, at six months 14 (41.2%) had no pain, 11 (32.4%) had mild pain, nine (26.5%) had moderate pain, zero patients had severe pain and at 12 months 19 (55.9%) had no pain, nine (26.5%) had mild pain, six (17.6%) had moderate pain, but none of our patients had severe pain. The pain at operation decreased significantly to that at six months (*p* < 0.0001) and 12 months (*p* < 0.0001).

Fourteen patients (41.2%) were working and 20 (58.8%) were not working or were students at the time of the screening. Nine of the patients (64.3%) working before the operation returned to their work.

Based on the prolonged hospitalization and preventive interventions, there were four (11.8%) not device-related serious treatment emergent adverse events (Table 2). One patient required screw removal seven months postoperatively due to screw protrusion. Other patient had malposition of the ankle requiring reoperation nine months postoperatively. One patient who had previously undergone a skin flap operation in the wound incision area developed deep infection requiring revision and musculocutaneous flap transfer. There were two aseptic drainages of β-TCP granules which were classified as device-related adverse events, which were harmless and self-limiting (Table 2).

**Table 2:** Safety results

Adverse event	β-TCP - NBP % (*n*)	List of events for β-TCP - NBP
Serious treatment emergent adverse events	11.8% (4)	1 deep wound infection, 1 malposition, 1 screw protrusion, 1 pneumonia
Device-related treatment emergent adverse events	5.9% (2)	2 aseptic drainages of β-TCP granules
Complications associated with surgical procedure	26.5% (9)	1 malposition, 1 screw protrusion, 4 wound infections, 1 pneumonia, 2 urinary tract infections
Minor complications or infections associated with surgical procedure	8.8% (3)	3 superficial wound infections

## Discussion

The primary endpoint, assessed by CT at six months, indicated that 85.3% of the joints achieved a fusion rate of ≥ 25%. This is somewhat higher than the 78.7% average fusion rate reported in the recent review by Leslie et al. [4]. Their evaluation included 26 articles on ankle and hindfoot fusion using autografts and various bone substitute materials, though the materials and methods were quite heterogeneous. In contrast, our fusion rate using the same criteria is comparable to the 82% observed in the autograft group and in the group of a product with same type of composition in the study by Krause et al. [17]. Overall, with a 25% threshold, the performance of β-TCP - NBP appears to be on par with autograft, the gold standard, in ankle and hindfoot arthrodesis, as supported by the literature [11, 17]. Thus, β-TCP - NBP offers a viable alternative to autograft.

Higher fusion rate thresholds have also been considered. In the studies by DiGiovanni et al. [11] and Daniels et al. [12], the fusion rate with a 50% threshold for the autograft group was 62.6% at six months, whereas our rate was slightly lower at 52.9%. However, the appropriateness of the 50% threshold for determining fusion and surgical success has been questioned because it is clinically too challenging [4].

Computer tomography has revolutionized the diagnosis of the fusion and non-union of the ankle and hindfoot [18, 19]. Different timepoints for CT have been used but six months seems to be the most common and was selected in this study as the primary endpoint like in the majority of comparable studies [4, 10, 11, 17–19]. There is no consensus concerning the threshold criteria for fusion rate by CT. However, the fusion rate ≥ 25% is the most commonly applied [4, 17–21].

The rate of serious treatment emergent adverse events not related to the device was in our study 11.8%, which is comparable to the literature (14.8–15.0%) [11, 12]. However, rate of device-related adverse events was slightly higher in our study (5.9%) compared to the literature (3.6–4.2%) [11, 12]. The two device-related adverse events were aseptic drainage of β -TCP granules which is a common phenomenon with ceramic bone graft substitutes and are not regarded as significant [11, 22]. For example, even when these have been measured, they have not been considered in the results [11]. To avoid this harmless self-limiting complication, the implant material should be introduced carefully into the fusion area and should not contact the skin.

Rate of complications associated with surgical procedure in our study (26.5%) is in line with the study by DiGiovanni et al. (30.3%) [11]. Overall, the safety profile in our study seems to be comparable to that in the autograft group in that study [11]. In summary, complications in our study were routine postoperative complications commonly associated with foot and ankle surgery.

The fusion procedure demonstrated its benefit by significantly alleviating pain, as measured by clinical scales and the VAS, and by enhancing patient function, as assessed by the AOFAS score, in individuals suffering from debilitating osteoarthritis of the ankle and hindfoot. Compared to the autograft group in the study by DiGiovanni et al. [11], our VAS pain scores at rest were slightly lower at six and twelve months (baseline values were not reported), whereas the VAS pain scores during weight-bearing were marginally higher. The AOFAS scores at six and twelve months were approximately five points higher in the autograft group of DiGiovanni et al.‘s study [11], though these differences were not statistically significant. These discrepancies may be attributed to the fact that the AOFAS scores utilized in our study were not validated for language.

Nine of the patients (64.3%) working before the operation returned to their work. Among the five patients who did not return to work, only one had a plausible reason, with a fusion rate of less than 25% showing faint osseous bridging. The remaining four patients demonstrated successful fusions and were practically pain-free. However, no underlying cause for not returning to work was identified in three cases, and the employment status of one patient was unrecorded. This means that returning to work is multifactorial parameter and does not directly relate to healing of the fusion.

Although tobacco users and users of medications affecting the skeleton or immune system were excluded, our results are sufficiently generalizable considering gender, age and diseases. The limitation of this study was the lack of a control group.

The common thought is that performance of ceramic bone substitutes is not sufficient for ankle and hindfoot fusion, as they do not perform on the similar level as autograft [23]. It was assumed that incorporation of tricalcium phosphate with osteoinductive substance would increase the healing potential to the desired level. In support of this assumption, this study demonstrated that β-TCP - NBP is a notable alternative for a bone graft substitute in ankle and hindfoot arthrodesis.

## Data Availability

The data that support the findings for this study are available to other researchers from the corresponding author upon reasonable request.
